# How physical activity opportunities seized by adolescents differ between Europe and the Pacific Islands: the example of France and New Caledonia

**DOI:** 10.12688/openreseurope.18385.1

**Published:** 2024-10-15

**Authors:** Thibaut Derigny, Marie-Jeanne Urvoy, Guillaume Wattelez, Pierre-Yves Leroux, Paul Zongo, Christophe Schnitzler, Olivier Galy, Francois Potdevin

**Affiliations:** 1 Université de Pau et des Pays de l’Adour, E2S UPPA, MEPS, Anglet, France, University of Pau and Pays de l’Adour, Pau, Nouvelle-Aquitaine, France; 2 Interdisciplinary Laboratory for Research in Education, University of New Caledonia, Nouméa, New Caledonia, University of New Caledonia, Noumea, South Province, New Caledonia; 3 Teaching and Research Unit in Physical Education and Sport, Canton de Vaud, (HEP-VD), University of Teacher Education, 1007 Lausanne, Switzerland, University of Lausanne, Lausanne, Vaud, Switzerland; 4 Unité de Recherche ‘Sport et sciences sociales’, UR 1342, Université de Strasbourg, France, University of Strasbourg, Strasbourg, Grand Est, France; 5 Univ. Lille, Univ. Artois, Univ. Littoral Côte d’Opale, ULR 7369 – URePSSS – Unité de Recherche Pluridisciplinaire Sport Santé Société, F-59000 Lille, France, University of Lille, Lille, Hauts-de-France, France

**Keywords:** Health, Geography, Culture, Accelerometery, Moderate and vigorous physical activity, Ecological framework

## Abstract

**Background:**

France (FR) and New Caledonia (NC) are both French territories, one in Western Europe, the other as part of the Pacific Island Countries and Territories (PICTs). Despite schooling in similar educational systems, FR and NC adolescents develop distinct relationships with physical activity, which is influenced by the geographical-cultural and symbolic structures of their respective societies. This study explored the distribution of physical activity according to geographical culture and opportunity-temporal dimensions.

**Methods:**

Participants were randomly selected, with individual (boys vs. girls), spatial (rural vs. urban), and geographical (FR vs. NC) stratifications. Accelerometers GT3X (ActiGraph ^TM^, Pensacola FL, USA) and daily logbooks were used to measure the physical activity intensity and opportunities during the week.

**Results:**

A total of 156 participants were included in this study. A significant effect was found in moderate to vigorous physical activity (MVPA) intensity with the geographical-cultural dimension; participants living in FR were more likely to engage in MVPA, especially in five opportunities: school, supervised leisure, home, school breaks, and transport. For both FR and NC adolescents, physical education lessons had the highest MVPA.

**Conclusion:**

This study showed that MVPA differed in the same national educational system according to geographical culture. Physical education lessons could catch the challenge of an “opportunity education” (opportunities are defined as temporal invitations to engage in PA) by opening the door to two particular opportunities: supervised leisure and active transport.

## Plain language summary

France and New Caledonia are both French territories, one in Western Europe, the other as part of the Pacific Island Countries and Territories. Despite schooling in similar educational systems, FR and NC adolescents develop distinct relationships with Physical Activity (PA). This study explored the distribution of PA according to geographical culture and the opportunity which structured the day. 156 participants worn an accelerometers and daily logbooks to measure the PA intensity and opportunities during the week. A significant effect was found in PA intensity with the geographical-cultural dimension: participants living in FR were more likely to engage in PA, especially in five opportunities: school, supervised leisure, home, school breaks, and transport. For both FR and NC adolescents, physical education lessons had the highest PA. Physical education lessons could collaborate with two other opportunities by opening the door of two particular opportunities: supervised leisure and active transport. This study highlights four points relevant to promoting healthy and sustainable active behaviour:

–  Living in New Caledonia decreases the odds of engaging in MVPA compared to France.

–  New Caledonian and French adolescents distinguish their MVPA in active transport and supervised leisure.

–  Physical education lessons provide the highest MVPA for both territories.

–  Physical education lessons might educate students about catching other MVPA opportunities.

## Introduction

Participation in daily physical activity (PA) is crucial for good health and well-being ( Ekelund *et al.*, 2019). However, 80% of adolescents from Western countries (such as mainland France: FR) do not reach the recommended 60 minutes of PA per day ( Bull *et al.*, 2020). Despite similar educational systems, approximately 50% of the adolescents of New Caledonia (NC), a French member of the Pacific Island Countries and Territories (PICTs), meet this recommendation ( Frayon *et al.*, 2020). Inactive behaviour leads to 80% of non-communicable diseases ( GBD, 2024), and adolescents in FR and NC are strongly exposed to these threats ( Swinburn *et al.*, 2019; Verdot *et al.*, 2022).

The literature shows that lifestyle and daily PA engagement are culturally and geographically oriented ( Ferrari *et al.*, 2020; Lera-López & Marco, 2018), which might lead to significant differences between FR and NC. NC is more affected by events and lifestyle transitions than FR: the 17 million PICT descendants were involved in farming activities, such as fishing and hunting, which are PA vectors ( Coyne *et al.*, 2000; Galy *et al.*, 2022; Wattelez *et al.*, 2024), although climatic and economic changes have had a major impact on behavioural changes ( McLennan & Ulijaszek, 2015). Exploring the physical inactivity of adolescents in two European Union territories with different ecosystems might indicate that the promotion of PA is culturally influenced.

Geographical culture profoundly influences adolescents’ relationships with PA, as suggested by the perspectives of various anthropologists. According to Mauss (1925), cultural practices, including PA, are “ *total social facts*” encompassing multiple dimensions of collective life. In FR, PA is often institutionalized through school or sports clubs, emphasizing competition and performance. Mead (1928) demonstrated that youth socialization varies according to the cultural context. In NC, PA may be more connected to traditional and community activities, reflecting the values of mutual aid and connection to nature. Geertz (1973) suggested that these practices are symbolic and reflect the meaning of each society. Thus, FR and NC adolescents develop distinct relationships with PA, which are influenced by the geographical and cultural symbolic structures of their respective societies. However, in these multicultural systems, these two territories have a similar education system with the same strategy of health education through PA.

To understand FR and NC behavioural differences, the bioecological model ( Bronfenbrenner & Morris, 2006) has been recommended in the literature ( Araújo & Davids, 2009). Adapted to the particularities of PA ( Bauman *et al.*, 2012) and explored in the context of PICTs ( Peralta *et al.*, 2022), it explains behavioural changes through the interaction of four systems: onto-system (e.g., sex), environmental system (e.g., space), global system (e.g., geography and culture), and chrono-system (e.g., time). For example, a lack of physical competence ( Wattelez *et al.*, 2021) is one of the main barriers to PA in an individual system. According to Zongo *et al.* (2017), only 20% of NC adolescents living in rural areas (spatial, as an environmental dimension) comply with PA recommendations, compared to 45% in urban areas. Martins *et al.* (2021) also highlighted the importance of access to sports installations (the spatial dimension). Few studies have considered the interaction of these three dimensions (individual, spatial, and cultural) with the temporal evolution of societies, despite the lack of time being the main barrier recognized in the international literature ( Duffey *et al.*, 2021).

Focusing on the temporal dimension, Telford *et al.* (2013) identified Wednesday as the most active day for adolescents, and Remmers *et al.* (2019) identified the early evening hours as most favourable for PA. However, this chronological approach does not consider the interaction process between the individual and the three targeted ecological dimensions ( Olds *et al.*, 2012). Elias (1997) advocated the investigation of the time during which opportunities are seized ( *kairos*) rather than the time that passes in the daily schedule ( *chronos*). Perceived as affordances, opportunities are thus defined as temporal invitations to engage in PA. Studies have identified eleven opportunities for structuring daily patterns ( Klinker *et al.*, 2014; Remmers *et al.*, 2020).

In FR, Derigny *et al.* (2022) identified the PA opportunities for adolescents and highlighted that the most active adolescents are those who seize a large number of opportunities rather than those who target specific opportunities. In NC, the rhythms observed during a typical school day differ from those observed in FR. Indeed, adolescents in both rural and urban areas wake up very early as schools begin between 7 and 7.30 am. When the school transport time is factored in, sleep duration is de facto reduced, with wake-up times between 5 and 5.30 am − even before 5 am for some of these families ( Wattelez *et al.*, 2024).

In NC, dynamics, which are induced by transitions, are the result of a lack of understanding of local habits and culture when new ways of PA engagement are being introduced. The current challenge is not to reproduce these findings with the promotion of PA, which, although it seems to be better than in FR, also seems to be deteriorating. Despite these individual, spatial, cultural, and temporal differences, the educational policies implemented in FR and NC are similar and do not appear to take into account the local particularities, although this is a strong recommendation ( Swinburn *et al.*, 2019). Exploring these four dimensions (individual, spatial, cultural, and temporal) could constitute a unique approach that may influence PA practices by distinguishing EU territories according to Western countries and PICTs.

Using objective (accelerometers for sedentary behaviour [SB], light PA [LPA] and moderate to vigorous PA [MVPA]) and subjective (daily logbooks for opportunities) measurements, this study explored the distribution of active behaviour according to individual, spatial, cultural and temporal dimensions. The two main hypotheses are that (i) FR adolescents have a lower level of active behaviour than NC adolescents, but (ii) opportunities in an urban lifestyle are more numerous but less seized upon than those in a rural lifestyle, where PA is more condensed into a few opportunities. These differences are all the more pronounced in NC than in FR as the cultural dimension is more traditional.

## Methods

### Participants

**Figure 1.**
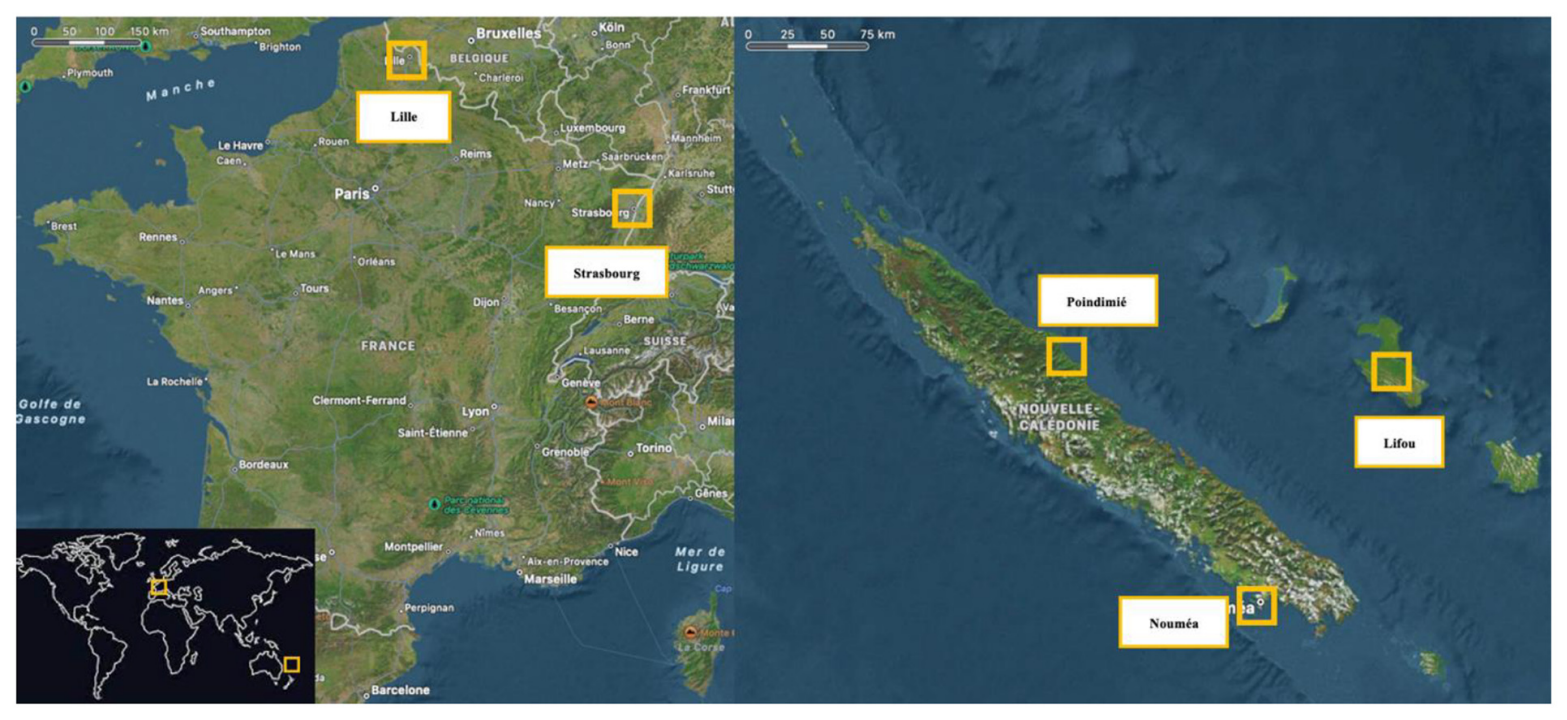
Geographical position of the cities included in the study Left: France; Right: New Caledonia

Two hundred and seventy participants in the last year of secondary school were selected in FR (n=160) and NC (n=110) between September 2021 and July 2022. Following the degree of urbanization, schools were selected based on their location, varying from rural (>2000) to urban (<2000, INSEE, 2020). In NC, the specificity of traditional bush and tribal lifestyles was grouped under the common rural category. As shown in Figure 1, data were collected in secondary schools in the region of Lille (urban and rural, Hauts-de-France, FR), Strasbourg (urban and rural, Grand-Est, FR), Nouméa (urban, Province Sud, NC), Poindimié (rural, Province Nord, NC) and Wé (rural, Lifou îles Loyauté, NC).

Participants were randomly selected, with individual (sex, boys vs. girls), spatial (rural vs. urban) and cultural (FR vs. NC) stratifications (more or less 50% of participants in each category). The two inclusion criteria were (i) agreement to wear the accelerometer for more than three weekdays and one weekend day for at least 10 hours per day ( Vanhelst, 2019) and (ii) agreement to complete more than 70% of the logbook (for reconstruction of daily opportunities). For all recruited participants, sociodemographic data were collected including age, sex, height, and weight with a tape and scale balance.

Before taking part in the study, written consent was obtained from the participants and parents or caregivers if they were under 18 years old (age of majority in FR and NC). This study was conducted according to the guidelines of the Declaration of Helsinki (2013) and was approved twice by the Ethics Committee of the University of Lille (2020-418-S82): firstly, for the data collection in FR (approval April, 28 ^th^ 2020), and secondly, for the data collection in NC (approval June, 27 ^th^ 2022). The Consultative Ethics Committee of New Caledonia was also required (CCE 2018-06 01, approval June, 1 ^st^ 2018) and the NCIF (National Committee for Informatics and Freedoms number 2020-037, approval April, 28 ^th^ 2020) from the University of Lille, was necessary to assure data protection.

### Measures

PA data were collected using a GT3X+ accelerometer (ActiGraph ^TM^, Pensacola FL, USA) at a sampling rate of 30 Hz. Participants wore the accelerometers on their preferential hip, fastened with an elastic belt for seven consecutive days ( Vanhelst, 2019). Data were reintegrated using a 10-s epoch. Troiano *et al.* (2008) wear time validation algorithm was applied, associating non-wear time to all periods >60 minutes of consecutive counting at zero. According to Freedson *et al.* (1998), the cut-offs used to define the intensity levels of SB and MVPA in the ActiGraph output were respectively a maximum of 100 counts.min ^-1^ and a minimum of 2295 counts.min ^-1^. Sleep time was excluded. This assessment tool is a proprietary research instrument that we have obtained a copyright license.

Daily social activities from participants were obtained from the logbooks to reconstruct the opportunities for the week. Diary logbook were based on pre-existing studies including eleven typical social opportunities ( De Baere *et al.*, 2015; Derigny *et al.*, 2022; Klinker *et al.*, 2014): (i) autonomous leisure, (ii) home, (iii) homework, (iv) job, (v) meal, (vi) PE lesson, (vii) recess, (viii) relax, (ix) school, (x) supervised leisure and (xi) transport. All the time not reconstructed were classed under “other”, the category which was used as an exclusion criterion: this category could represent no more than 30% of the logbook for the participant to be included. More precision on each opportunity can be found in Derigny *et al.* (2022).

Data from the accelerometers and logbooks were combined in the Actilife software (proprietary research instrument that we have obtained a copyright license) and compared following individual (boys vs. girls), spatial (urban vs. rural), temporal (eleven opportunities) and cultural (NC vs. FR) dimensions. Opportunity ratio values were calculated for SB, LPA and MVPA by dividing the time spent at each intensity by the duration of the same opportunity ( Derigny *et al.*, 2024).

### Statistics

Statistical analyses were conducted with R software (version 4.3.2). Data and residuals of age, body mass index (BMI) and time spent in SB, LPA and MVPA were tested for normality, interdependence and homoscedasticity using Shapiro-Wilks, Levene and Jarque-Bera tests. As these preliminary conditions were not met, non-parametric tests on median values and interquartile ranges were used. The threshold of statistical significance was set at 5% ( *p*<.05).

First, Pearson Chi-squared tests and Krustal-Wallis tests were used to detect effects on age, sex, BMI, accelerometer wear time, and SB, LPA and MVPA according to the culture (FR vs. NC). Then, a general linear regression model, reporting the odds ratio and 95% confidence intervals, was used to examine the effects of individual, spatial and cultural dimensions. The interaction with the temporal dimension was explored with a series of Kruskal-Wallis tests. Analysis was conducted on the “opportunity ratio” (the time in SB or PA divided by the total time spent in the opportunity) as it is not possible to compare time variables if their durations were not the same. The effect sizes were determined using partial eta-squared values (η _p_^2^) and their confidence intervals, which are interpreted as follows: η _p_^2^>0.01: small; η _p_^2^>0.06: medium; and η _p_^2^>0.14: large ( Lakens, 2013).

## Results

### Sample characteristics

Descriptive statistics of the whole sample and according to the geographic culture are presented in Table 1. One hundred and fifty-six participants were included in the study (58% of the volunteers). The two groups had homogeneous characteristics regarding age, sex, and spatial (place of residence) dimensions. The BMI was significantly higher for participants in NC compared to FR (21.6 vs. 23.6, *p*<0.004). According to the protocol, the two groups wore the accelerometers equally (FR vs. NC: 5,710 vs. 5,557, p=0.6).

**Table 1.** Participant characteristics included in the study.

	Overall (n = 156)	France (n = 86)	New Caledonia (n = 70)	*Geographic culture effect*	
				*p*-value	*ηp ^2^*
Age 1	17.0 ± 1.58	16.8 ± 2.04	17.3 ± 0.68	0.2	0.06 [0.02; 0.17]
Body mass index	22.2 (20.2, 25.5)	21.6 (19.5, 24.6)	23.6 (21.0, 25.9)	**0.004**	0.39 [0.11; 0.84]
GT3X wear time 2	5,691 (5,118, 6,274)	5,710 (5,224, 6,191)	5,557 (4,525, 6,451)	0.6	0.78 [0.47; 0.78]
**Sex**				0.3	0.01 [0.01; 0.05]
Women	109 (70%)	46 (66%)	63 (73%)	-	-
Men	47 (30%)	24 (34%)	23 (27%)	-	-
**Spatial**				0.4	0.06 [0.01; 0.05]
Rural	62 (40%)	37 (43%)	25 (36%)	-	-
Urban	94 (60%)	45 (64%)	94 (60%)	-	-
**Physical activity behaviour**					
SB	77 % (74, 80)	78 % (74, 81)	77 % (74, 80)	0.2	0.01 [0.01; 0.04]
LPA	16 % (14, 20)	15 % (13, 17)	18 % (15, 20)	**< 0.001**	0.01 [0.01; 0.03]
MVPA	5.8 % (4.5, 8.0)	6.3 % (5, 8.6)	5.1 % (3.8, 6.9)	**0.002**	0.02 [0.01; 0.08]

*Notes. Data are presented in median (IQR)*

^1^: mean ± standard deviation

^2^: minutes.week ^-1^; bold: significant effect

The accelerometric data show that participants in the two cultures had the same level of SB (78% vs. 77%, *p*=0.2), but participants in NC had a higher level of LPA (15% vs. 18%, *p*<0.001) and a lower level of MVPA (6.3% vs. 5.1%, *p*=0.002) than those in FR.

### Relationships between active behaviour and sex, spatial and cultural dimensions

Table 2 presents the three successive models of multivariate regressions. No effect of sex or spatial dimension was identified for SB, LPA or MVPA. A significant effect was found for MVPA for the geographical cultural dimension: participants living in NC were less likely to engage in MVPA (OR=0.67, *p*=0.036). No effect of culture was shown for SB and LPA.

**Table 2.** Odds ratio for SB, LPA and MVPA according to sex, spatial and geographical cultural dimensions.

	SB			LPA			MVPA		
	*OR*	*95%CI*	*p-value*	*OR*	*95%CI*	*p-value*	*OR*	*95%CI*	*p-value*
**Sex**									
Women	-	-	-	-	-	-	-	-	-
Men	0.98	0.78 – 1.25	0.889	0.99	0.74 – 1.31	0.927	1.34	0.9 – 1.95	0.139
**Spatial**									
Rural	-	-	-	-	-	-	-	-	-
Urban	1.17	0.93 – 1.46	0.173	0.84	0.64 – 1.09	0.179	1.15	0.79 – 1.69	0.475
**Geographical cultural**									
FR	-	-	-	-	-	-	-	-	-
NC	0.98	0.78 – 1.21	0.823	1.04	0.80 – 1.35	0.755	**0.67**	**0.45 – 0.97**	**0.036**

Notes. OR: odds ratio; IC: confidence interval; bold: significant effect.

### Interaction between opportunities and cultural dimension

**Figure 2.**
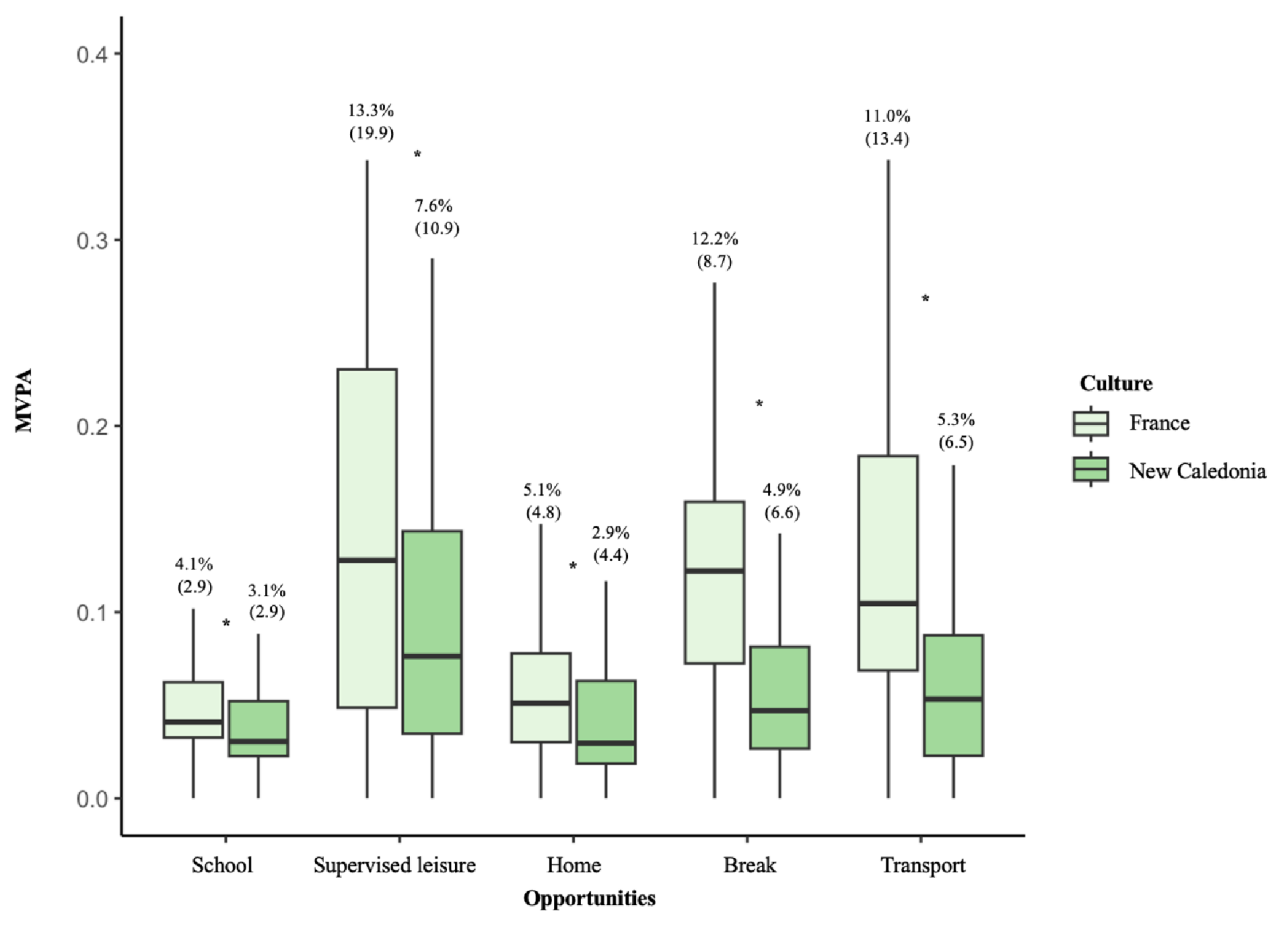
Opportunities of MVPA with significant cultural effect. Data are presented with median (IQR); *: significant differences according to the cultural dimension.

**Figure 3.**
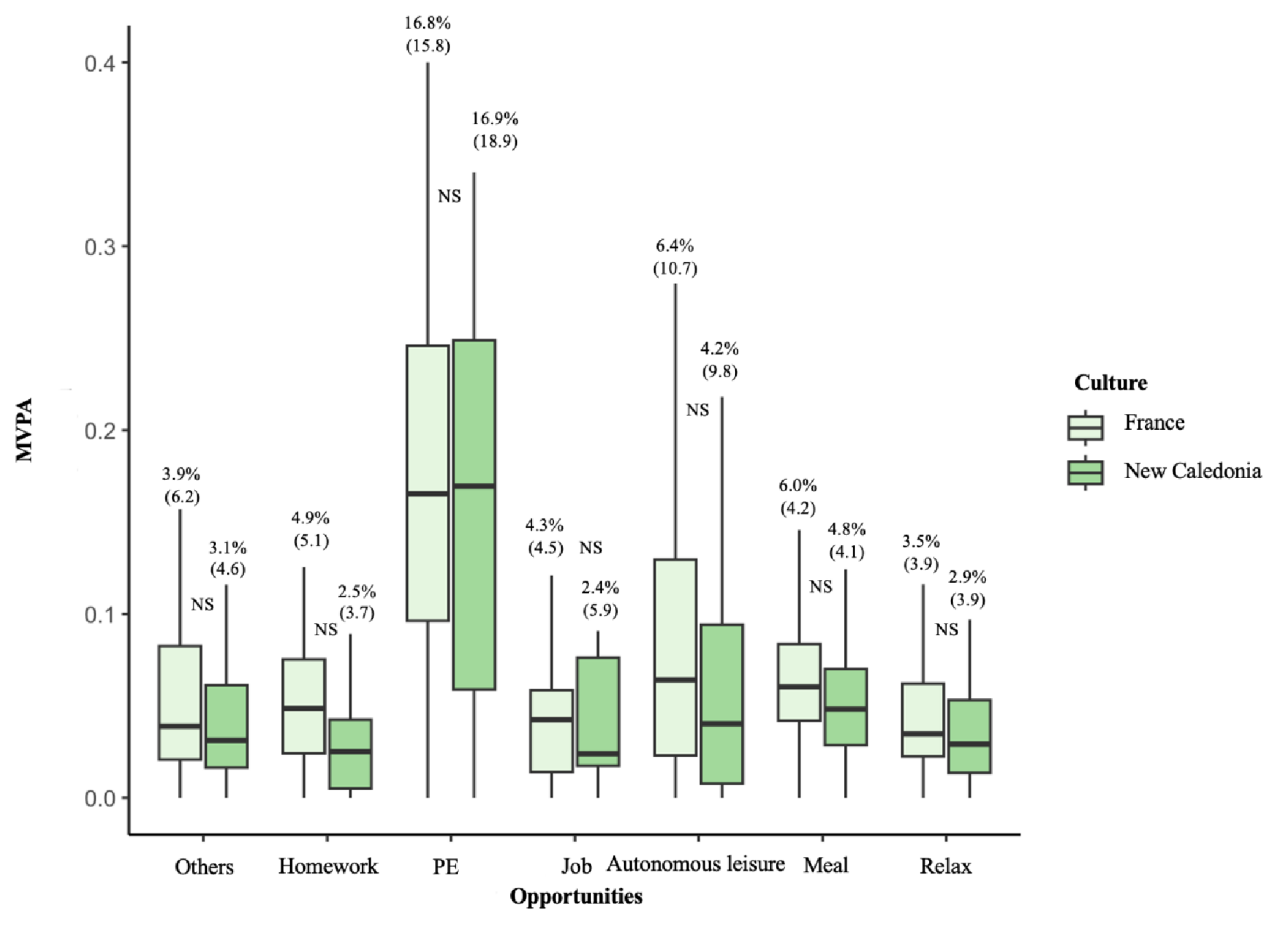
Opportunities of MVPA with non-significant cultural effect. Data are presented with median (IQR); PE: physical education lesson; NS: not-significant.

MVPA The interaction between MVPA and the geographical cultural dimension was explored with a series of Kruskal-Wallis tests (because it was the only one with a significant effect in the regression model). Five opportunities for MVPA differed significantly between FR and NC, as presented in Figure 2: school (4.1 vs. 3.1, *p*=0.006), supervised leisure (13.3 vs. 7.6, *p*=0.025), home (5.1 vs. 2.9, *p*=0.004), school break (12.2 vs. 4.8, *p*<0.001) and transport (11.1 vs. 5.3, *p*<0.001). The MVPA of the seven other opportunities (including “others” as well as all times non-reported) did not differ significantly between FR and NC and are presented in Figure 3: others (3.9 vs. 3.1, *p*=0. 657), homework (4.9 vs. 2.5, *p*=0.064), physical education (16.8 vs. 16.9, *p*=0.351), job (4.3 vs. 2.4, *p*=0.824), autonomous leisure (6.4 vs. 4.2, *p*=0.086), meal (6.0 vs. 4.8, *p*<0.068) and relax (3.5 vs. 2.9, *p*=0.076). For both the FR and NC cultures, the opportunity of physical education lesson was the one with the highest level of MVPA (16.7 vs. 16.9, *p*=0.35).

## Discussion

This study showed that individual, spatial, cultural and temporal dimensions had an impact on engagement in MVPA opportunities within the same national educational system. We found that: (i) participants living in FR were more likely to engage in MVPA, (ii) five opportunities differed significantly between FR and NC, always in favour of the MVPA behaviours of FR (school, supervised leisure, home, school break and transport, and (iii) physical education lessons provide the best MVPA opportunities for both FR and NC adolescents.

### What is the extent of the PA differences between these two French territories?

Results showed that participants living in FR were more likely to engage in MVPA compared to those living in NC. This study is the first to compare PA behaviours based on European and Pacific lifestyles using a multi-level approach (individual, space, culture, and time) and similar tools (accelerometers and diaries). Nedjar-Guerre *et al.* (2023) showed that NC adolescents have high rates of sedentary behaviour and screen time, especially those living in urban areas. Wattelez *et al.* (2024) highlighted the short sleep durations in NC. Similar associations have been identified among French adolescents ( Messaadi *et al.*, 2020), but the methodologies of the FR and NC studies do not allow for reliable comparisons. This result is original in that it contradicts the current literature and reveals the importance of taking a closer look at the complex problem of insufficient PA.

### Which opportunities are most likely to foster MVPA in all adolescents?

MVPA differed significantly for five opportunities between FR and NC adolescents, always in favour of FR: school, school break, supervised leisure, home, and transport. Although van Sluijs *et al.* (2021) suggested that school is the best place to promote and educate about sustainable PA. The difference in MVPA engagement suggests more PA promotions in NC schools. Break facilities might be an avenue worth exploring to promote PA ( Lemberg *et al.*, 2023). School-based PA has been shown to help improve academic results ( Redondo-Flórez *et al.*, 2022; Solberg *et al.*, 2021) and well-being at school ( Joing *et al.*, 2020; Smedegaard *et al.*, 2016).

Supervised leisure activities should also be further explored, but neither the grey nor the research literature provides information on the number of sports clubs in NC and the affiliations of adolescents. Practices may be evolving in NC, taking inspiration from the “Promotion de la Santé au sein des Clubs Sportifs” (PROSCeSS) program, which has been recognized as a convincing action by French Public Health (2024) for the promotion of health within sports clubs ( Van Hoye *et al.*, 2021). Differences in the PA engagement at home may be explained by differences in lifestyles. Being active at home with fitness equipment is more in keeping with a Western lifestyle, whereas the rhythm in NC is focused on outdoor activities, which can be conducive to PA ( Frayon *et al.*, 2020; Frayon *et al.*, 2021; Zongo *et al.*, 2017). Finally, with regard to transport, Schönbach *et al.* (2020) demonstrated the need for education coupled with environmental planning in order to encourage young people to engage in PA. Cycle paths in NC appear to be less developed than those in FR. Investing the space-geography of these opportunities seems to be a necessary perspective in NC.

Physical education lessons did not differ between FR and NC adolescents, but exhibited the highest opportunity ratio values, thus representing the best opportunity to engage all adolescents in MVPA. These results confirmed those reported in the systematic review of Silva *et al.* (2022) who showed that adolescents engaged in more MVPA on the day where physical education lesson took place. However, the opportunity ratio, between 16.7% (FR) and 16.9 (NC), was rather low and confirmed the results of Derigny *et al.* (2022) and the systematic review of Burson *et al.* (2021), who respectively showed that only 22% and 31% of PE lessons are dedicated to MVPA.

### Is the physical education opportunity catching up to the challenge of PA promotion?

Results showed that the opportunity to engage in MVPA was the highest in the physical education lessons for both territories. Physical education lessons are also able to overcome MVPA territorial differences, when a cultural effect was observed. However, in the NC context, it was even more difficult for physical education lessons to educate students to take advantage of environmental opportunities, since the other opportunities were less often caught by the NC adolescents. Can PE achieve its ambition to promote PA? We think that it depends on the goal: (i) if the main goal is to encourage students to immediately engage in PA during the physical education lesson, the answer is yes, but (ii) if the main goal is to encourage students to make a long-term commitment to PA within one’s environment, the answer is no.

The opportunity of physical education lessons could invest an “opportunity education”. Drawing on systemic and ecological approaches borrowed from public health sciences, education for PA opportunities requires collaboration in which those responsible for each opportunity contribute to the promotion of PA. This theoretical framework has been shown to be effective ( Hynynen *et al.*, 2016). The systemic promotion of PA can be centralised during mandatory physical education lessons. In concrete terms, working with those responsible for the spatial environment to build cycle paths to schools is necessary to promote PA among adolescents in the context of active transport. At the same time, active transport education is needed in physical education lessons. This concept has led to a redefinition of PA ( Piggin, 2020) as “ *people moving, acting and performing within culturally specific spaces and contexts, and influenced by a unique set of interests, emotions, ideas, instructions and relationships*”.

Although the literature has been rich regarding pedagogical methods to increase adolescents’ MVPA and reduce SB ( Rodrigo-Sanjoaquín *et al.*, 2022), the discrepancy between the FR and NC participants suggests the need to (i) develop specific teaching strategies according to each PA profile in PE ( Turcotte *et al.*, 2021), (ii) increase the total time of PE per week, and (iii) educate students about the environmental opportunities. These issues are essential in a European context where two territories with different cultures share physical education curricula, training and sports installations.

### Strengths and limitations

The strength of this study is the measurement of PA by accelerometery and its limitation is the documentation of social occasions by a self-reported questionnaire. The category of “other” referred to all opportunities when participants were awake that could not be reconstructed by the logbook. Three limitations should be noted. First, the number of participants in the subgroups (individual, space and culture dimensions) was small, but this was balanced by the finding that the regression models were significant. Second, sociodemographic status was not taken into account, whereas it might have had an impact on PA. Last, the lack of granularity regarding the cultural communities within NC and FR, which are very different, was a shortcoming, since culture is here perceived only in its geographical dimension (FR vs. NC). Future studies are needed to explore the trajectories of these active behaviours in each opportunity when these adults become adults. The use of a mixed method with qualitative interviews of participants from both cultures would allow a better understanding of the gaps from the point of view of the participants’ experiences.

## Conclusion

The objective of this study was to examine how the level of PA differs between French adolescents from two territories with different cultures: Western Europe (FR) and PICT (NC), according to individual (boys vs. girls), space (urban vs. rural), and time (all the opportunities of the day) dimensions. The opportunity-based approach shows that PA has extrinsic (“building an opportune environment”) and intrinsic (“catching opportunities”) values that are correlated with adolescent commitment. This study suggests that the physical education opportunity is able to overcome territorial differences, but, in the NC context, this challenge of an “opportunity education” in physical education lessons does not seem to have yet been raised.

To address this issue, public policies in NC should focus on promoting traditional physical activities that align with local cultural values. Implementing community-based programs that encourage outdoor and nature-related activities can help reconnect adolescents with their cultural heritage. In addition, educational campaigns that highlight the benefits of integrating traditional practices with modern physical activities can create a balanced approach. These policies should also address urban planning to provide safe and accessible spaces for physical activity, particularly in urban areas where sedentary behaviour is more prevalent.

This study suggests the following recommendations: (i) initiate an epidemiological monitoring of PA in NC in order to set up an observatory, (ii) maintaining PA analysis from a bioecological point of view, and (iii) integrate these elements into the proposed education in order to reverse any trend towards a decline in PA.

## Ethics and consent

This study is part of two global projects (Family farming, lifestyle, and health in the Pacific [FALAH], led by the University of New-Caledonia; Distribution of Physical Activity [DAP], led by the University of Lille, France). This study was conducted according to the guidelines of the Declaration of Helsinki (2013) and was approved twice by the Ethics Committee of the University of Lille (2020-418-S82): firstly, for the data collection in FR (approval April, 28 ^th^ 2020), and secondly, for the data collection in NC (approval June, 27 ^th^ 2022). The Consultative Ethics Committee of New Caledonia was also required (CCE 2018-06 01, approval June, 1 ^st^ 2018) and the NCIF (National Committee for Informatics and Freedoms number 2020-037, approval April, 28 ^th^ 2020), from the University of Lille, was necessary to assure data protection. Before entering the study, written consent was obtained from the participants and parents or caregivers if they were under 18 years old.

## Data Availability

Zenodo: How physical activity opportunities seized by adolescents differ between Europe and the Pacific Islands: the example of France and New Caledonia. https://doi.org/10.5281/zenodo.13150401 ( Derigny *et al.*, 2024) The project contains the following underlying data: Data_NCFR: xlsx files with data on culture, spatial, city, opportunity, anthropometric, sedentary and active behaviour. Zenodo: How physical activity opportunities seized by adolescents differ between Europe and the Pacific Islands: the example of France and New Caledonia. https://doi.org/10.5281/zenodo.13150401 ( Derigny *et al.*, 2024) The project contains the following underlying data: Script_NCFR: R files with the statistical analysis; Logbook_NCFR: pdf file with an example of a logbook for collecting data on daily opportunities. Consent_NCFR: pdf file with the consent form for participants. Data are available under the terms of the Creative Commons Attribution 4.0 International (CC-BY 4.0).
