# Supplementary material for: How physical activity opportunities seized by adolescents differ between Europe and the Pacific Islands: the example of France and New Caledonia
Source: Open Res Eur. 2024 Oct 15;4:217. [Version 1] doi: 10.12688/openreseurope.18385.1 (PMC11751613; doi:10.12688/openreseurope.18385.1)
Supplement: Peer review history

# Reviewer report: Reviewer response for version 1

**DOI:** 10.21956/openreseurope.19865.r49146

**Published:** 2025-01-21

**Authors:** Fitria Dwi Andriyani

**Affiliations:** 1 Department of Sports Education, Universitas Negeri Yogyakarta (Yogyakarta State University), Yogyakarta, Indonesia

The study aimed to investigate the differences in PA levels between adolescents in France and New Caledonia with different cultures by including individual, space, and time/opportunity dimensions. 

 Overall, the article is well presented. The use of both device-based and self-report measurement strengthens the study. 

 However, I have some questions and recommendations for the authors below. 

 Introduction

 1. "In FR, PA is often institutionalized through school or sports clubs, emphasizing competition and performance." (Please clarify references to support this statement). 

 2. "In NC, PA may be more connected to traditional and community activities, reflecting the values of mutual aid and connection to nature." (What is the basis of this statement?)

 3. "Thus, FR and NC adolescents develop distinct relationships with PA, which are influenced by the geographical and cultural symbolic structures of their respective societies". (More supporting sentences are needed to support this statement - to be more convincing)

 4. "In NC, the rhythms observed during a typical school day differ from those observed in FR. Indeed, adolescents in both rural and urban areas wake up very early as schools begin between 7 and 7.30 am. When the school transport time is factored in, sleep duration is de facto reduced, with wake-up times between 5 and 5.30 am − even before 5 am for some of these families ( Wattelez *et al.* , 2024)." (The authors explained the observation in NC. What about observation in FR? The authors need to provide a comparable explanation). 

 5. "In NC, dynamics, which are induced by transitions, are the result of a lack of understanding of local habits and culture when new ways of PA engagement are being introduced. The current challenge is not to reproduce these findings with the promotion of PA, which, although it seems to be better than in FR, also seems to be deteriorating. Despite these individual, spatial, cultural, and temporal differences, the educational policies implemented in FR and NC are similar and do not appear to take into account the local particularities, although this is a strong recommendation ( Swinburn *et al.* , 2019). Exploring these four dimensions (individual, spatial, cultural, and temporal) could constitute a unique approach that may influence PA practices by distinguishing EU territories according to Western countries and PICTs." (The authors need to restructure this paragraph. For the current version, I know that you aim to explain the importance of studying the four dimensions (individual, spatial, cultural, and temporal). However the transition from the previous paragraph to this idea was not smooth yet, but confusing).

 5. The authors may need to consider revising the use of the term "objective" vs "subjective" measurement. I understand that these terms are widely used. However, we can't say that accelerometers are fully *objective* as the way to process the data involved many *subjective* decision, e.g., determining cut-off point, non-wear time, etc. I would prefer to use "device-based measurements" rather than objective measurements, and "self-report measurements" rather than subjective measurements. 

 6. " The two main hypotheses are that (i) FR adolescents have a lower level of active behaviour than NC adolescents, *but *(ii) opportunities in an urban lifestyle are more numerous but less seized upon than those in a rural lifestyle, where PA is more condensed into a few opportunities." (Why the authors use " *but"*? needs clarification).

 7. Please consider reducing the use of abbreviations throughout the document (FR, NC, PICTs).  The reader may find it not too convenient to read such a document.  

 Method

 1. Please clarify why the authors used cut-off points following Freedson *et al.* (1998), and the wear time validation following Troiano et al. (2008).

 Result

 1. The BMI was significantly higher for participants in NC compared to FR (21.6 vs. 23.6, *p*<0.004) (I think you mentioned it in the wrong order, it should be 23.6 vs 21.6?)

 Discussion 

 1. "The difference in MVPA engagement suggests more PA promotions in NC schools." (Do you think PA level can be increased by PA promotion only? Please elaborate more on how PA promotion may increase PA level). 

 2. "The systemic promotion of PA can be centralised during mandatory physical education lessons." (Is physical education lesson not mandatory in FR and NC?)

 3. " In concrete terms, working with those responsible for the spatial environment to build cycle paths to schools is necessary to promote PA among adolescents in the context of active transport." (Is there any safety issue regarding active transport in FR and NC?)

 4. Your hypotheses as mentioned in the introduction: "The two main hypotheses are that (i) FR adolescents have a lower level of active behaviour than NC adolescents, but (ii) opportunities in an urban lifestyle are more numerous but less seized upon than those in a rural lifestyle, where PA is more condensed into a few opportunities." (please clarify why you have such hypotheses and how they relate to your finding. In which part do you discuss your second hypothesis?).

If applicable, is the statistical analysis and its interpretation appropriate?

I cannot comment. A qualified statistician is required.

Is the study design appropriate and is the work technically sound?

Yes

Is the work clearly and accurately presented and does it engage with the current literature?

Yes

Are the conclusions drawn adequately supported by the results?

Yes

Are sufficient details of methods and analysis provided to allow replication by others?

Yes

Are all the source data and materials underlying the results available?

Yes

Reviewer Expertise:

physical activity, sedentary behaviour

I confirm that I have read this submission and believe that I have an appropriate level of expertise to confirm that it is of an acceptable scientific standard.

# Reviewer report: Reviewer response for version 1

**DOI:** 10.21956/openreseurope.19865.r45833

**Published:** 2024-11-21

**Authors:** Nuttanun Siriaporn

**Affiliations:** 1 Universite Cote d'Azur, Nice, Provence-Alpes-Côte d'Azur, France

This article compares physical activity opportunities among adolescents between France and New Caledonia. With similar educational systems, the study compared genders, urban/rural classification, and geographical culture. The article found that physical education classes provided the highest MVPA for both populations and that it has the potential to educate and increase PA through supervised leisure and active transport.

 The study is straight-forward and well-written. The results are well-supported by other literature.

 Specific comments:

 - In the second section of the Discussion, "Which opportunities are most likely to foster MVPA in all adolescents?" The sentence, "The difference in MVPA engagement suggests more PA promotions in NC schools." is not very clear. Is the sentence saying that the difference is that there is more PA promotion in NC or that more PA promotion is needed?

 - In the same section as the previous, it was mentioned that NC may be evolving in supervised leisure activities due to PROSCeSS program, which is supported by French Public Health. Is there support that this program could be implemented in NC? If not, it seems a bit speculative.

If applicable, is the statistical analysis and its interpretation appropriate?

I cannot comment. A qualified statistician is required.

Is the study design appropriate and is the work technically sound?

Yes

Is the work clearly and accurately presented and does it engage with the current literature?

Yes

Are the conclusions drawn adequately supported by the results?

Yes

Are sufficient details of methods and analysis provided to allow replication by others?

Yes

Are all the source data and materials underlying the results available?

Yes

Reviewer Expertise:

public health; active transport; health promotion; health behavior

I confirm that I have read this submission and believe that I have an appropriate level of expertise to confirm that it is of an acceptable scientific standard.
